# Positive childhood experiences and burnout among medical students: the role of adaptive emotion regulation as a mediator

**DOI:** 10.3389/fpsyg.2025.1707795

**Published:** 2026-01-08

**Authors:** Rachel Lloyd, Tina Izad, Michael Nazmifar, Lauren Walkon, Changiz Mohiyeddini

**Affiliations:** Oakland University William Beaumont School of Medicine, Rochester, MI, United States

**Keywords:** adaptive emotion regulation, positive childhood experiences, burnout, mediation analysis, power analysis

## Abstract

Burnout is a state of chronic exhaustion caused by excessive stress. Medical trainees are highly vulnerable to burnout, which can negatively affect their well-being. Empirical evidence from Bethell et al. demonstrates that positive childhood experiences reduce adult depression and anxiety, while Gross’s process model suggests that adaptive emotion regulation strategies buffer against stress-related outcomes. Building upon these frameworks, positive childhood experiences (PCE) and adaptive (positive) emotion regulation (ER) are thought to be protective against burnout, but the mechanisms which mediate these effects are poorly understood. This study aimed to explore whether adaptive ER mediates the relationship between PCE and burnout among medical students. Participants completed well-established and validated measurements of PCEs (Benevolent Childhood Experiences scale), adaptive ER (Cognitive Emotion Regulation Questionnaire short version), and burnout (Maslach Burnout Inventory). Our results indicate that adaptive ER mediates the relationship between PCEs and burnout, with a moderate indirect effect of PCEs on burnout (*β* = −0.31, accounting for approximately 67% of the total effect). These findings suggest that targeted intervention programs aiming to strengthen adaptive ER could help protect medical students against burnout. However, limitations include the cross-sectional design precluding casual inference, reliance on self-reported data, and recruitment from a single institution with predominantly female participants, which may limit generalizability to other medical schools.

## 1 Introduction

Burnout is a highly prevalent mental health issue among medical students. Burnout is defined by three characteristics in the ICD-11: feelings of energy depletion or exhaustion, increased mental distance, negativity, or cynicism related to one’s job, and reduced professional efficacy (World Health Organization, n.d.). It is estimated that at least half of all medical students will experience burnout during their medical education (IsHak et al., 2013). Additionally, two key indicators of burnout, emotional exhaustion and cynicism, worsen as students progress through medical education (Kilic et al., 2021). In addition to poor academic performance (Madigan and Curran, 2021), burnout is associated with emotional distress and depression, substance use disorders, and suicide (Amir et al., 2018). Rates of depressive symptoms and suicidal ideation are strikingly high among medical students, with one systematic review indicating the prevalence of depressive symptoms and suicidal ideation at 27.2 and 11.1%, respectively (Rotenstein et al., 2016). The mental health crisis among medical students is clear, but factors protecting against burnout and the associated mental health conditions have yet to be established.

Among the factors hypothesized to protect against burnout are PCEs and adaptive ER (Martín-Brufau et al., 2020; Brown et al., 2022). PCEs are defined by the CDC as the experience of having safe, stable, nurturing relationships and environments in childhood (Sege et al., 2024). These experiences are conceptualized as factors supporting the development of resilience, ultimately helping to buffer against later adversity. PCEs are known to promote healthy child development and adult mental health, as well as reduce the prevalence of adult health risk behaviors (Bethell et al., 2019; Graupensperger et al., 2023). Research demonstrates that individuals with higher PCEs show greater psychological flexibility, stronger support networks, and more adaptive coping strategies in adulthood. Bethell et al. (2019) found that PCEs were associated with reduced depression and anxiety symptoms, even among adults who experienced childhood adversity. Similarly, Narayan et al. (2018) demonstrated that PCEs predicted lower stress reactivity and better mental health outcomes among pregnant women with adverse childhood experiences. Additionally, Kocatürk and Çiçek (2023) found that higher levels of PCEs predicted more positive psychosocial functioning, including higher self-esteem and resilience among adults. These findings suggest that early positive experiences are linked to lower psychopathology in adults, though the mechanisms underlying the association remain unclear.

ER is defined as the process of observing, evaluating, and adjusting emotional experiences and responses (Thompson, 1994). ER relies on the cognitive evaluation of emotionally significant cues, followed by the usage of strategies that modulate the individual’s emotional response to the cue (Garnefski and Kraaij, 2018; Ochsner and Gross, 2008). Strategies such as positive appraisal or refocusing are considered adaptive and aid in effective adjustment to challenging situations (Garnefski et al., 2001). In contrast, strategies such as self-blame, rumination, or catastrophizing are considered maladaptive and contribute to intense emotional reactions to difficult situations (Garnefski and Kraaij, 2018; Sætren et al., 2025). In a group of healthcare employees working in a long-term care facility for the elderly, adaptive emotion regulation, such as cognitive reappraisal, was associated with reduced anxiety and stress among employees (Wobeto et al., 2023). This evidence highlights the idea that adaptive ER serves as a pathway through which individuals maintain psychological wellbeing, reinforcing the central argument of the present study.

While previous studies have examined the long-term effects of adverse childhood experiences, our study is one of the few to investigate PCEs. To our knowledge, the relationship between PCEs and burnout has not been evaluated. The role of ER and dysregulation as a primary variable related to mental health has been studied extensively, but its potential role as a mediating factor has not been established. Additionally, the possible mediating effect of adaptive ER between PCEs and burnout has yet to be studied among the medical student population. The findings also carry practical significance, as identifying adaptive ER as a mediator highlights an actionable target for medical education programs seeking to reduce burnout through curricular or wellness-based interventions.

Therefore, we investigated following hypotheses:

*H1*: PCEs will be negatively associated with burnout.

*H2*: PCEs will be positively associated with adaptive ER.

*H3*: Adaptive ER will be negatively associated with burnout.

*H4*: Adaptive ER will mediate the association between PCEs and burnout.

The link between PCEs, adaptive ER, and burnout can be understood through multiple theories. Gross’s process model of emotion regulation provides a theoretical framework for this connection, as it emphasizes how adaptive cognitive strategies shape emotional responses to stressors (Gross, 2002). In this model, early positive experiences may strengthen regulatory pathways that buffer against burnout in adulthood. Fredrickson’s Broaden-and-Build theory of positive emotions posits that early positive emotions expand cognitive capacities, which strengthen adaptive emotion regulation and resilience (Fredrickson, 2004). These mechanisms are thought to improve the odds of coping later in life, which may reduce burnout. Additionally, Cicchetti and Masten address the impact of early childhood environments on future risk of psychopathologies (Cicchetti, 2010). They emphasize the importance of a supportive health environment to promote improved coping skills and an improved ability to tolerate stress. In the context of our research, it echoes the role of PCEs in helping to protect individuals from burnout. Furthermore, Masten suggests resilience is more ubiquitous than it is perceived (Masten, 2001). Her research proposes that normal coping mechanisms serve a role in producing resilience amongst individuals. Concern develops when these coping mechanisms are disrupted, typically due to significant adversity that makes the ability to be resilient become more difficult. PCEs are thought to help foster these normative resilience skills into adulthood. Lastly, the Job Demands-Resources (JD-R) model explains how workplace stress and burnout arise from an imbalance between job demands and resources (Demerouti et al., 2001). When demands exceed resources, individuals experience energy depletion, ultimately leading to burnout. The model further suggests that personal resources, such as adaptive ER, can serve to protect against burnout.

## 2 Methods

### 2.1 Study design

A single-arm, non-interventional, cross-sectional design was used to investigate whether adaptive ER mediates the association between PCEs and burnout in medical students.

### 2.2 Power analysis

We conducted a Monte Carlo power simulation (5,000 repetitions) in R using lavaan package to determine the necessary sample size to examine the proposed mediation model. In contrast to traditional power calculations based on Cohen’s d, Monte Carlo power simulation are adequate to test mediation models. The model was parameterized with standardized medium effect sizes, based on Cohen’s conventions and consistent with the strong, well-established relationships between PCEs, adaptive ER, and burnout in the literature. The simulation indicated that a sample size between 114 and 132 participants would be optimal to achieve a statistical power between 91 and 95% for detecting the indirect effect (*α* = 0.05). Our final sample of *N* = 124 falls within this optimal range, confirming the analysis was adequately powered to test the proposed model.

### 2.3 Sample and procedure

Participants were recruited by emailing the survey to the Oakland University William Beaumont (OUWB) School of Medicine listserv three times from October 2024 to May 2025. Inclusion and exclusion criteria were verified by asking participants to report their age and year of schooling, and because the survey link was only distributed via the school listserv, only individuals meeting these criteria were able to access and complete the survey. Listwise deletion was applied to surveys in which participants completed only minimal information, such as the first few demographic questions, without providing responses to the remaining portions of the survey. Information regarding non-respondents was not available, so potential differences between respondents and non-respondents could not be assessed.

A total of 148 medical students participated in this study. Of these, 124 participants (83.8%) completed the survey, while 24 students (16.2%) with missing data were excluded using listwise deletion. No data imputation was conducted.

For gender identity, the majority of respondents identified as female (*n* = 89; 71.8%), followed by male (*n* = 34; 27.4%), and non-binary (*n* = 1; 0.8%), based on valid responses. This gender distribution aligns with national trends in medical school enrollment, which increasingly show higher representation of women in medical education. The mean age of participants was 24.5 years (SD = 3.26).

Regarding year of education, the largest proportion of participants were second-year medical students (M2), comprising 46.8% of valid responses (*n* = 58). First-year students (M1) represented 32.3% (*n* = 40), while third-year (M3) and fourth-year (M4) students accounted for 12.1% (*n* = 15) and 8.9% (*n* = 11), respectively. This distribution suggests that lower-year students were more likely to participate in the survey, which may reflect availability, engagement with wellness initiatives, or timing of the recruitment relative to curricular demands.

### 2.4 Ethical considerations

This study was conducted in accordance with the principles outlined in the Declaration of Helsinki and approved by the Institutional Review Board (IRB) of Oakland University (IRB-FY2024-362). The data collection was anonymous and no identifiable information was recorded.

### 2.5 Analytical approach

Prior to analysis, the dataset was screened for missing values, outliers, and violations of normality assumption. The mediation model was specified with PER as the mediator of the relationship between PCEs and burnout. Full mediation was hypothesized because prior research suggests that PCEs promote adaptive emotion regulation, which in turn reduces burnout, implying that the protective effect of PCEs on burnout operates through PER. However, alternative models, such as partial mediation or reciprocal relationships between PER and burnout, were considered. These models are reasonable alternatives to the full mediation model, emphasizing the need for future studies to clarify the directionality and magnitude of these associations.

To explore the mediation hypothesis, we used hierarchical regression analysis using bootstrapping with 5,000 resamples. Statistical significance was determined based on 95% bias-corrected confidence intervals. Prior to conducting regression analyses, standard diagnostic checks were performed to assess model assumptions. Residual plots were examined for linearity and homoscedasticity, and variance inflation factors (VIFs) were calculated to assess multicollinearity. No violations of assumptions were detected, and all VIFs were below 2.0, indicating low multicollinearity. All statistical analyses were conducted using R (Version 4.4.0; R Core Team, 2024). All variables were standardized prior to analysis to ensure comparability across measures (Demerouti et al., 2001) which facilitates direct comparison of regression coefficients. This is particularly useful when variables are measured on different metric ranges, as unstandardized coefficients may otherwise be difficult to interpret in relative magnitude (Gelman, 2008).

### 2.6 Instruments

The questionnaire contained socio-demographic information and measurements of PCEs, adaptive ER, and burnout.

PCEs were measured using the Benevolent Childhood Experiences (BCE) Scale. The BCE scale has been validated across multiple populations, including ethnically diverse low-income pregnant women (Narayan et al., 2018) and parents of young children residing in an emergency homeless shelter (Merrick et al., 2019). BCE consists of 10 questions assessing experiences between birth and age 18 defined by perceived safety, security, and relational support. It also assesses positive and predictable characteristics of one’s daily life in childhood (e.g., regular meals, consistent bedtime) (Narayan et al., 2018). Participants answered “yes” or “no” to each item, indicating whether they had experienced the specified condition during childhood. Internal consistency of BCE was very high (*α* = 0.89) in this study. A higher score on the BCE indicates a higher quantity of positive childhood experiences.

Adaptive ER was measured using the Cognitive Emotion Regulation Questionnaire short version (CERQ-short), which assesses cognitive ER strategies, positive refocusing, planning, positive reappraisal, putting into perspective, and acceptance. Each subscale is measured by two items using a 5-point Likert scale ranges from “almost never” to “almost always.” A higher score on a particular subscale corresponds to a greater amount of time spent utilizing this cognitive approach for ER (Garnefski and Kraaij, 2006). The CERQ-short has demonstrated strong psychometric properties in both clinical and non-clinical populations and is widely used in academic stress research.

The inter-item correlations for each scale were highly significant (rs < 0.01), ranging from *r* = 0.74 (Refocus on planning) to *r* = 0.91 (acceptance), indicating strong internal consistency. To assess adaptive ER, we computed a composite score by averaging the five adaptive CERQ subscales. This approach aligns with prior work demonstrating that adaptive strategies, such as positive reappraisal and acceptance, are associated with lower symptoms across multiple psychopathologies (Aldao et al., 2010). Internal consistency for the composite score was high (*α* = 0.79), supporting its reliability (Garnefski and Kraaij, 2006).

Burnout was measured using the Maslach Burnout Inventory (MBI). This survey consists of 22 questions assessing the three key components of burnout, including emotional exhaustion (EE), depersonalization (DP), and reduced personal accomplishment (PA). The MBI uses a 7-point Likert scale ranging from “never” to “everyday.” Internal consistency for the MBI was acceptable in this sample (α = 0.77). A higher score demonstrates higher burnout strain (Brady et al., 2020).

## 3 Results

### 3.1 Descriptive statistics

Descriptive statistics indicated moderate levels of burnout among participants (*M* = 3.93, SD = 1.76), with a relatively high mean for positive childhood experiences (*M* = 0.82, SD = 0.26) and moderately high levels of adaptive ER (*M* = 3.39, SD = 0.93). These values suggest that while students reported relatively favorable childhood environments and moderate ER capabilities, burnout symptoms were still prevalent, indicating the multifactorial nature of burnout in the setting of medical education (Table 1).

**Table 1:** The descriptive results.

	Burnout	Adaptive ER	PCEs
Burnout			
Adaptive emotion regulating (Adaptive ER)	−0.737		
Positive childhood experiences (PCEs)	−0.468	0.472	
Mean (SD)	3.93 (1.76)	3.39 (0.93)	0.82 (0.26)

*N* = 124.

### 3.2 Correlation analysis

Preliminary analyses showed that age, gender, and year of medical education were not significantly correlated with PCEs, adaptive ER, or burnout (all ps > 0.10). Therefore, these demographic variables were not included as covariates in the mediation analyses. However, burnout was negatively associated with both PCEs (*r* = −0.468, *p* < 0.001) and adaptive ER (*r* = −0.737, *p* < 0.001). Furthermore, PCEs were positively correlated with adaptive ER (*r* = 0.472, *p* < 0.001).

### 3.3 Mediation analysis using hierarchical regression analysis

**Figure 1:**
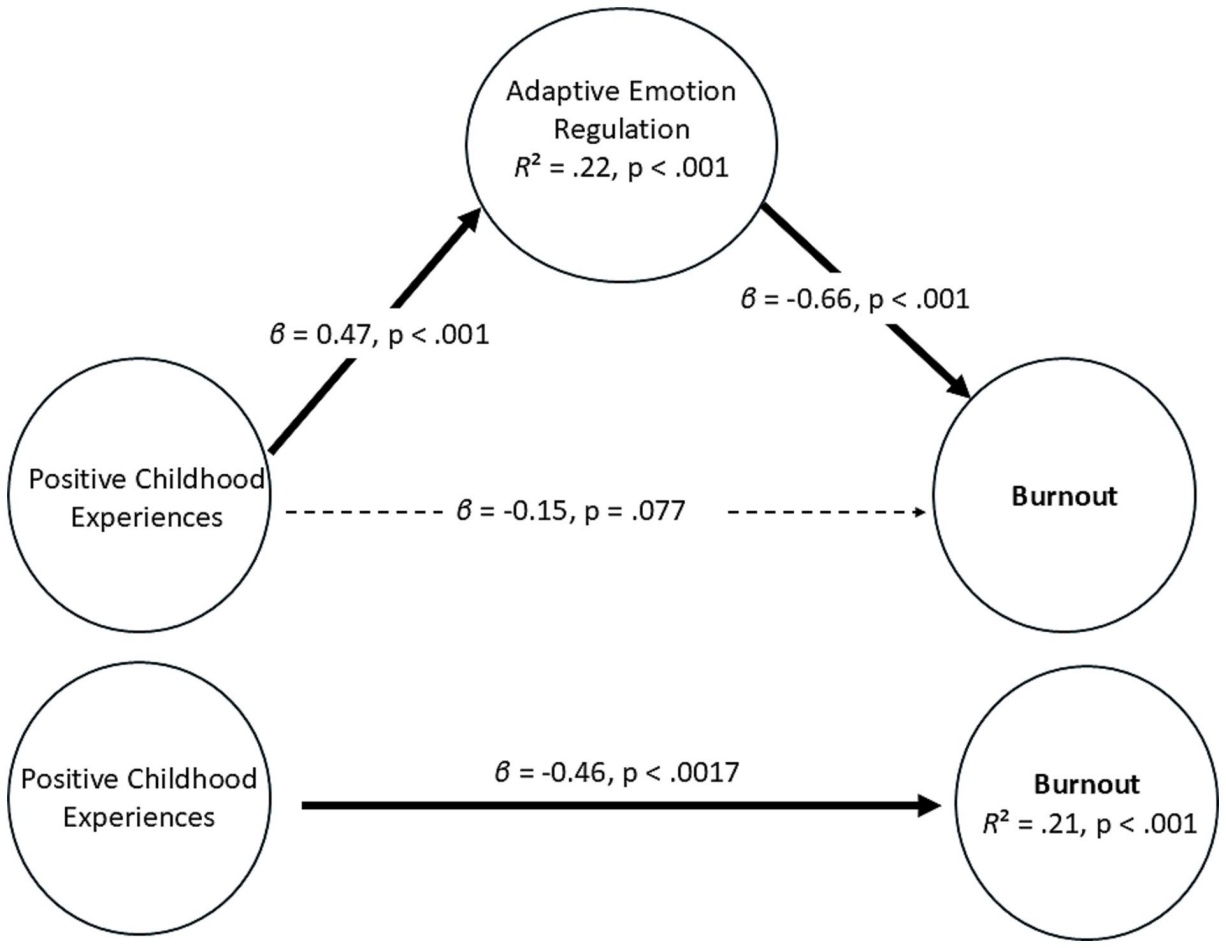
Mediation model of positive childhood experiences, positive emotion regulation, and burnout.

The results of the mediation analysis are presented in Figure 1 and detailed below. Standardized regression coefficients are reported.

The results show that PCEs had a significant positive association with adaptive ER, (*β* = 0.47, *p* < 0.001). The model accounted for a significant portion of the variance in the mediator (*R*^2^ = 0.22, *p* < 0.001). In addition, adaptive ER had a significant negative association with burnout after controlling for PCEs, (*β* = −0.66, *p* < 0.001). Furthermore, the direct link between PCEs and burnout was reduced and no longer significant when the mediator was included in the model (*β* = −0.15, *p* = 0.077), suggesting a total mediation of the association between PCEs and burnout by adaptive ER. For comparison, the unmediated direct link between PCEs and burnout was (*β* = −0.46, *p* < 0.001). Furthermore, the analysis also revealed a significant indirect association between PCEs and burnout through adaptive ER (*β* = −0.31, *b* = −2.05; 95% CI [−3.48, −0.93]).

## 4 Discussion

The observed correlations are consistent with H1, H2, and H3. As hypothesized PCEs were negatively associated with burnout (H1) and positively associated with adaptive ER (H2). Adaptive ER was negatively associated with burnout (H3). These hypotheses were also supported by the results of the mediation analysis. Furthermore, the results of the mediation are consistent with H4. Adaptive ER mediated the association between PCEs and burnout to the extent that the direct link between PCEs and burnout decreases significantly to be non-significant.

The significant association between PCEs, adaptive ER, and burnout aligns with existing literature suggesting that early positive relational and environmental factors can foster resilience and effective ER strategies later in life. For instance, studies have demonstrated that PCEs are associated with lower rates of adult psychopathology, including depression and anxiety (Kocatürk and Çiçek, 2023; Gross, 2002). These positive experiences likely support adaptive cognitive processes, such as reappraisal and acceptance, early in life, which are crucial for regulating emotions in high-stress situations. Gross’s process model of ER proposes that individuals engage in a variety of strategies to influence which emotions they have, when they have them, and how they experience and express these emotions (Gross, 2002). In this model, adaptive strategies such as positive reappraisal, cognitive reframing, and acceptance have been associated with lower psychological distress and improved well-being (Garnefski et al., 2001; Aldao et al., 2010). Fredrickson’s Broaden-and-Build theory of positive emotions proposes that early positive emotions expand cognitive capacities, which strengthen adaptive emotion regulation and resilience (Fredrickson, 2004). These mechanisms are thought to improve the odds of adaptive coping later in life. Additionally, the Job Demands-Resources (JD-R) model proposes that burnout arise from an imbalance between job demands and resources (Demerouti et al., 2001). This model suggests that personal resources, such as adaptive ER, can serve to protect against burnout. In the context of medical education, where students are routinely exposed to high-pressure environments, long working hours, and emotionally challenging situations, the ability to regulate emotions effectively becomes critical.

The clinical implications of these results are significant. First, they suggest that ER strategies can be a viable target for interventions aimed at reducing burnout in medical students. Existing intervention programs, such as mindfulness-based stress reduction (MBSR) and cognitive-behavioral therapy (CBT), already incorporate elements of ER and have shown efficacy in reducing burnout and improving mental health in healthcare (Shapiro et al., 2005; Regehr et al., 2013). Medical schools might consider integrating structured ER training into their curricula, potentially as part of existing wellness or professional development programs. Such interventions could include workshops on cognitive restructuring, reflective practices, stress inoculation training, and peer support groups.

Second, the finding that PCEs contribute to burnout protection indirectly through ER abilities has implications for student support services. While early life experiences cannot be modified retrospectively, understanding their influence can help inform individualized support strategies. Students with fewer PCEs may be at greater risk for developing burnout due to a weaker foundation in ER. In such cases, early identification and tailored interventions can provide critical support. Interventions could include resilience and ER workshops that teach strategies such as cognitive reappraisal and stress management, mindfulness-based training to enhance adaptive coping, and targeted counseling services utilizing cognitive or dialectical behavior therapy for students demonstrating low adaptive ER skills.

Medical schools have a vital role in cultivating institutional environments that promote ER and psychological resilience. This includes fostering a culture of psychological safety, where students feel comfortable being vulnerable and seeking help without fear of judgment or academic consequences. Faculty mentorship programs, regular discussions about emotional challenges, and normalized access to mental health professionals can contribute to this culture. Furthermore, reducing systemic contributors to burnout, such as curriculum overload, excessive assessment frequency, and lack of flexibility, can significantly alleviate the environmental pressures that overwhelm students’ coping abilities.

## 5 Limitations

One limitation of this study is its cross-sectional design, which excludes causal inference. Although mediation analysis suggests a directional relationship, longitudinal data are necessary to establish temporal precedence. Another limitation is the reliance on self-report instruments, which are subject to bias and may not fully capture the complexity of burnout or ER in real-world contexts. Social desirability bias, in particular, may lead students to underreport symptoms of burnout or overreport positive attributes. Furthermore, because listwise deletion was used, findings may be biased if missing data were not random. Additionally, the sample was drawn from a single medical school, which may limit generalizability to other institutions with differing cultures, support systems, or academic demands. Although the OUWB student body is predominantly female, the 71.8% female response rate may further constrain the applicability of findings to more gender-balanced populations. In addition, the OUWB school culture is generally collaborative rather than highly competitive, which may foster supportive interactions and enhance ER skills relative to more competitive environments. Furthermore, the curriculum structure could have affected responses, as many participants were M2s completing the survey under the stress of preparing for Step 1, which may have temporarily heightened burnout or influenced perceived ER abilities. While this study focused on adaptive ER, the absence of maladaptive ER measures limits a full understanding of emotional regulation processes. Future research should consider dual-process models incorporating both adaptive and maladaptive strategies to more comprehensively evaluate ER dynamics.

## 6 Implications and future directions

Despite these limitations, the study makes a meaningful contribution to the literature on medical student mental health. By identifying ER as a mediator between PCEs and burnout, this study advances theoretical understanding of how early protective factors translate into resilience in adulthood. Methodologically, the use of validated scales strengthens confidence of these relationships. Collectively, these insights provide a framework for both individual-level interventions and systemic educational reform. As medical education continues to change, embedding ER training into curricula offers an effective strategy for building competent physicians who are resilient and capable of dealing with the challenges of their profession.

Beyond individual intervention, systemic culture change must occur. There is a pervasive “hidden curriculum” in medical education that glorifies self-sacrifice and emotional suppression. This culture not only discourages help-seeking behavior, but also implicitly devalues self-care. The data from this study support the counterargument: emotional health and the capacity for adaptive regulation are not distractions from medical training, but integral to a successful, sustainable practice in medical school and beyond. Burnout among physicians is associated with increased medical errors, lower patient satisfaction, and greater turnover rates. By addressing burnout early during medical training, institutions are not only supporting student wellness but also improving patient care. Medical education, therefore, must evolve to meet the needs of trainees, recognizing that mental and emotional competencies are as critical to medical practice as the understanding of medical conditions and treatments.

Moreover, future research should move beyond correlation and regression to incorporate mixed methods and longitudinal designs. Qualitative interviews can add to quantitative findings, providing insights into the lived experiences of students who have developed strong ER abilities despite adverse early life experiences. Longitudinal studies can establish whether interventions aimed at increasing ER skills result in lasting reductions in burnout over time and into residency and early clinical practice. Randomized controlled trials testing the effectiveness of ER-based interventions, such as Acceptance and Commitment Therapy (ACT), CBT, or Dialectical Behavior Therapy (DBT), could offer concrete, evidence-based recommendations for institutional adoption.

In sum, this study adds to growing evidence that medical student burnout is a complex, multifaceted issue. The findings that PCEs contribute to lower burnout primarily through their association with adaptive ER underscore the significant role of emotion regulation skills in medical training. While not all students arrive at medical school with the same emotion regulation skills, institutions have the responsibility to develop these skills through curriculum design, targeted interventions, and institutional culture. Incorporating evidence-based training on ER, medical institutions have the power to cultivate a generation of future physicians who are more emotionally resilient and, in turn, better prepared to care for themselves and their patients.

## Data Availability

The raw data supporting the conclusions of this article will be made available by the authors, without undue reservation.
